# Gender-Specific Trajectories of Conduct Problems from Ages 3 to 11

**DOI:** 10.1007/s10802-017-0379-1

**Published:** 2018-01-05

**Authors:** Leslie Morrison Gutman, Heather Joshi, Michael Parsonage, Ingrid Schoon

**Affiliations:** 1 University College London, 1-19 Torrington Place, London, WC1E 7HB UK; 2 UCL Institute of Education, 20 Bedford Way, London, WC1H 0AL UK; 3 Centre for Mental Health, 2d21, Technopark 90 London Rd, South Bank, London, SE1 6LN UK

**Keywords:** Conduct problems, Gender-specific trajectories, Group-based trajectories, Early childhood

## Abstract

Gender-specific pathways of conduct problems (CP) from toddlerhood have received little attention. Using a nationally representative sample of UK children born in 2000–2001 (6458 boys and 6340 girls), the current study (a) identified subgroups of CP pathways separately for boys and girls from ages 3 to 11 and (b) examined early precursors (pregnancy to 9 months) of these trajectories. Group-based trajectory models identified four distinct trajectories for both boys and girls: each characterized as ‘low’; ‘early-onset, desisting’; ‘early-onset, persistent’ and ‘school-onset’. This suggests that the taxonomic framework developed to conceptualise childhood-onset CP among males is also applicable to females, though needing some revision to capture heterogeneity identified during early and middle childhood. We also found significant precursors of the different trajectory groups with some variation by gender. Early socioeconomic deprivation was a significant risk factor of the early-onset pathways among both genders, but played no significant role for ‘school-onset’. Childhood-onset trajectories of boys, but not girls, were predicted by parenting attitudes and behaviour.

There has been a growing interest in the identification and prognosis of conduct problems (CP) in early childhood. CP refers to behaviours under the conduct-oppositional spectrum, including those that are defiant, antisocial and/or potentially harmful to others such as lying, stealing, physical aggression and rule-breaking (APA 2000). Researchers have posited that the age of onset of CP provides valuable insight into its causal mechanisms as well as developmental pathway and associated later outcomes. Children who exhibit elevated and persistent CP from early childhood are at greater risk of continued CP throughout middle childhood, adolescence and adulthood compared to those who first develop CP later (Odgers et al. 2008). Childhood-onset CP is also one of the strongest predictors of delinquency, antisocial behaviours and substance abuse in adolescence and adulthood (Moffitt 2006; Odgers et al. 2008). Clearly, understanding heterogeneity in developmental pathways of CP from early childhood is a high priority for intervention purposes.

Much of the earlier work on CP focused exclusively on boys, given their higher incidence of early and persistent CP, or neglected to examine gender differences in pathways of CP (see Brennan and Shaw 2013, for a review). More recent studies allowing for gender differences in CP have tended to model developmental trajectories for boys and girls together, rather than estimating gender-specific trajectories (e.g., Barker and Maughan 2009; Joussemet et al. 2008; Tremblay et al. 2005; Van Lier et al. 2005). Establishing aggregate patterns in trajectories may underestimate the percentage of females in the childhood-onset, persistent group since females may demonstrate relatively lower mean levels of CP compared to males. It may also mask pathways evident in one gender but not in the other (Brennan and Shaw 2013). An examination of gender-specific pathways illuminates the similarities and differences in the development of CP and its related antecedents, correlates and consequences in boys and girls.

There are few prospective longitudinal studies examining gender-specific CP developmental pathways of males and females from the youngest age at which early starters can be identified reliably—around 3 years (Brennan and Shaw 2013; Shaw, Hyde, & Brennan, 2012). Most studies of gender-specific CP trajectories have examined a limited duration, beginning at school entry or in adolescence. Yet, there is reason to suppose that the developmental pathways of CP diverge for boys and girls from early childhood (Zahn-Waxler et al. 2008). Some research, for example, suggests that most girls desist in the use of aggression from an earlier age than boys (Hay et al. 2000; Keenan and Shaw 1997). Studies of CP trajectories from school-age may fail to differentiate between children who desist and those who persist from an early age.

There is also limited information on predictors of gender-specific pathways of CP from early childhood, especially using representative samples. There are likely to be both biological and social reasons behind the higher levels of CP among boys than girls (Moffitt et al. 2008; Meier et al. 2011). For example, boys’ higher likelihood of delayed language development and emotional regulation problems coupled with differences in parents’ responses may place them at greater risk of early-onset, persistent CP than girls (Zahn-Waxler et al. 2008). Yet, there is little research identifying the earliest precursors that may predict developmental CP trajectories for girls, specifically. Information on pre- and post-natal risk factors would be helpful in tailoring interventions, which are now primarily based on research on early-starting boys (Shaw 2013). It would be particularly useful to distinguish early on between those girls who are likely to desist on their own accord and those who would expressly benefit from early intervention efforts.

This study, using a nationally representative sample of children in the UK, aims to fill these gaps in knowledge through (1) identification of gender-specific developmental pathways of CP from ages 3 to 11 and (2) examination of pre- and post-natal risk factors that differentiate these CP developmental pathways. We first review the theoretical framework for conceptualizing developmental pathways of childhood-onset CP and then discuss research on gender-specific trajectories of CP, before laying out our research questions and hypotheses.

## Theoretical Framework

A developmental-pathways approach can help us understand the mechanisms underlying the onset and continuity of CP (Frick 2006). In one such approach, Moffitt (2006) identified two patterns of anti-social behaviour in children based on age of onset and developmental course: childhood-onset and adolescent-onset CP. The first shows persistent and elevated levels of CP from childhood, continuing to exhibit antisocial behaviour in adolescence and adulthood (Moffitt et al. 2002). This pathway likely has a biological component, reinforced by the environment experienced (Moffitt et al. 2008). Adolescent-onset CP, on the other hand, manifests during adolescence, most likely due to rebelliousness which passes as young people mature (Moffitt 2006; Odgers et al. 2008).

Within the childhood-onset pathway of CP, research has suggested that further distinctions can be made in terms of developmental trajectory, aetiology and later outcomes (Frick and Viding 2009). Among those with childhood-onset CP, many do not show CP later in development, often termed as the childhood-limited or desisting group (Moffitt et al. 2008). Research has shown that boys who show desisting CP patterns may not become antisocial, but are more likely to be depressed and anxious in adulthood (Moffitt et al. 2002).

## Developmental Taxonomy of Females

Both developmental taxonomy theory and existing empirical research in support of it have been largely based on males, which led to previous doubts about whether the developmental taxonomy is equally applicable to both genders (e.g., Silverthorn and Frick 1999). More recent studies of CP trajectories have identified a small but significant proportion of females showing a childhood-onset, persistent trajectory, with a separate group of females exhibiting an adolescent-onset trajectory (e.g., Broidy et al. 2003; Lahey et al. 2006; Odgers et al. 2008). However, there is some evidence for a childhood-onset, desisting group of females. Where found, their pathway tends to be alongside rather than instead of, a childhood-onset, persisting pathway (Brennan and Shaw 2013).

A childhood-onset, persisting CP trajectory of females has been identified in most studies which have examined gender-specific trajectories of CP and its related constructs, including antisocial behaviour and physical aggression but not including co-morbid constructs such as inattention/hyperactivity or more broadly, externalizing symptoms, from childhood (up to age 7) to early adolescence (ages 10 to 13) (Broidy et al. 2003; Campbell et al. 2010; Côté et al. 2001; Fontaine et al. 2008; Harachi et al. 2006). In a multi-site study from three countries, Broidy et al. (2003) identified a small group of females in three of the sites (Quebec; Christchurch NZ; and the US) who followed a persistent trajectory of aggression from early childhood. However, in the Dunedin, NZ birth cohort, they did not find a childhood-onset, persistent group of females, but rather a childhood-onset, desisting group. Only one of these studies documented both a childhood-onset, persistent group and a childhood-onset, desisting group of females (Fontaine et al. 2008). Not surprisingly, studies examining gender-specific trajectories in this age range did not identify an adolescent-onset trajectory of CP for either boys or girls. Studies examining a longer period from childhood to adolescence have demonstrated childhood-onset, persistent and adolescent-onset trajectories for both genders (e.g., Bor et al. 2010; Bongers et al. 2004; Lahey et al. 2006).

This research base on gender-specific trajectories of CP from childhood to early adolescence has its limitations. First, none of these studies identified CP trajectories from early childhood. With most beginning at school-age (ages 6 or 7), they lack evidence which might capture early-onset (i.e., before school-age), desisting patterns. Second, these studies assessed aggregated reports of disruptive, antisocial and physically aggressive behaviours, rather than CP specifically. Research has shown that boys engage in more overt symptoms of CP such as physical aggression, but both genders engage in similar levels of covert symptoms such as lying and stealing (Tiet et al. 2001). A clinically meaningful measurement of CP, which includes both covert and overt forms of CP, may be better able to detect diverse developmental patterns, understanding the pathways to clinically diagnosable conduct disorder. Third, they relied on teacher-reports rather than parent-reports. It is arguable that teachers may have a more limited familiarity with a child’s development than parents, and in any case, teachers cannot provide reports before school-age. In this study, we provide new evidence through examination of a maternal-reported measure of CP covering the crucial pre-school period and transition into early adolescence.

## Early Risk Factors of CP Developmental Pathways

According to a developmental-pathways approach, there are different aetiologies, each involving distinct combinations of risk factors that operate in the development of CP trajectories (Frick 2006). Here we focus on the earliest precursors that may distinguish among childhood-onset CP trajectories. According to Moffitt (2006), the childhood-onset pathway may be explained, in part, by children’s neuropsychological deficits, difficult temperaments and poor verbal abilities. In addition, children on the childhood-onset trajectory often live in households characterised by family instability and financial hardship, family conflict and ineffective, negative parenting (Frick and Viding 2009; Shaw 2013). Childhood-onset CP has also been associated with a greater likelihood of being born to a teenage mother and exposed to maternal smoking during pregnancy (Barker and Maughan 2009; Maughan et al. 2004).

Regarding childhood-onset, persisting versus desisting patterns, most research has shown that these two pathways have the same early risk factors, including poor verbal abilities, difficult temperaments, maternal psychopathology and poor parenting (Moffitt 2006; Moffitt et al. 2008; Odgers et al. 2007, 2008). However, there is evidence suggesting that children in the persistent group experience higher levels of maternal psychopathology and poor parenting than children in the desisting group (Barker and Maughan 2009). Therefore, although children with these two pathways appear to share similar risk factors, their trajectories may be differentiated by the range and severity of these early risks.

In terms of gender differences in childhood-onset CP, research has generally found few consistent risk factors that are moderated by gender (Brennan and Shaw 2013; Murray et al. 2010). Rather, the same early risk factors have been found to be associated with childhood-onset CP in both genders (Barker and Maughan 2009; Côté et al. 2006; Lahey et al. 2006). It thus has been suggested that boys’ greater vulnerability to childhood-onset CP may be due their greater number of child risk factors (e.g., language delays and higher rates of impulsivity and inattention) rather than family risk factors compared with girls (Messer et al. 2006). Nevertheless, there is little information concerning whether early family and child risk factors are uniquely associated with childhood-onset CP pathways in one gender but not the other, as relatively few studies have examined the earliest predictors of gender-specific trajectories of CP (Brennan and Shaw 2013). In this study, we fill this knowledge gap by examining whether early risk factors shown to characterise childhood-onset CP discriminate among gender-specific CP trajectories from ages 3 to 11. These include familial factors − socio-economic disadvantage, single parenthood, smoking in pregnancy, maternal psychopathology, family conflict and poor parenting − and infant factors – delayed development and difficult temperament (Frick and Viding 2009; Moffitt et al. 2008).

## Current Study

Using the UK Millennium Cohort Study (MCS), the current study aims to (a) identify different subgroups of childhood-onset CP trajectories separately for boys and girls from ages 3 to 11 and (b) examine the early precursors (pregnancy to 9 months) of these trajectories. First, we examine gender-specific developmental trajectories of maternal-reported CP at ages 3, 5, 7 and 11. Unlike studies that examine gender differences in trajectories of CP where males and females are grouped together, this strategy allows distinct trajectories to emerge for each gender. This is important as pathways may be identified for one gender, but not for the other. Despite the advantages of estimating gender-specific trajectories, they may identify groups which are not clinically meaningful e.g., a ‘high’ trajectory female group that engages in relatively low levels of CP (Brennan and Shaw 2013). To remedy this, we used clinically meaningful age cut-offs based on national norms, which have been shown to predict strongly later conduct disorder (Meltzer et al. 2000). Second, we assess early precursors (including pregnancy) measured when infants were 9 months-old including both familial and child risk factors. We examine whether these (a) show significant gender differences in their means and (b) differentiate the gender-specific trajectories in both univariate and multivariate analyses.

The present study contributes towards a better understanding of gender heterogeneity in childhood-onset CP trajectories, especially during early childhood and the potential mechanisms of risk. Based on previous evidence, we expect to identify trajectories of CP with distinct aetiologies (Brennan and Shaw 2013). We expect that there will be parallel pathways of CP for boys and girls, with both genders showing a childhood-onset, persistent pathway and a low one. In line with Campbell et al. (2010) and Côté et al. (2001), however, we may find a childhood-onset, desisting trajectory for boys, but not for girls. We also predict that there may be gender differences in the prevalence of particular CP trajectories. As in Brennan and Shaw (2013), a lower percentage of girls is expected to be in the childhood-onset, persistent group and a higher percentage is expected to be in the low group compared to boys. We also expect differences in the early risk factors among childhood-onset trajectories of CP. As documented (e.g., Barker and Maughan 2009), we might find that children in the childhood-onset, persistent group experience the same early risk factors but in a higher dosage compared to those in the childhood-onset, desisting trajectory group. We make no firm predictions about gender differences in the early predictors, given the limited previous evidence using multivariate analyses.

## Method

### Study Sample

MCS is a nationwide longitudinal study following children born the UK between September 2000 and January 2002 (Joshi and Fitzsimons 2016). This survey has a complex clustered and disproportionately stratified design (Hansen 2014). The strata oversampled areas of high child poverty, minority ethnic populations and the three smaller countries of the UK. The initial sample was identified by the Child Benefit authorities who approached eligible families with information about the study asking if they wished to opt out. If they did not opt out, they were sent further information in advance of the interviewer visit at which they were asked for written confirmation that they wished to participate. This procedure was repeated at each follow-up data collection, between which families were sent mailings about some of the study’s findings, also shown on a website dedicated to participating families. The surveys were granted ethical clearance by National Health Service Multi-Centre Research Ethics Committees (MREC). For MCS1 this was the committee based in the South West, for MCS2 and MCS3 the London MREC and for MCS4 and MCS5 the Northern and Yorkshire MREC.

Families have been interviewed six times, with results available for this analysis from five of them. The first survey, MCS1 (child age 9 months) was in the field mainly in 2001–2002, data on age 3 (MCS2), age 5 (MCS3) and age 7 (MCS4) were collected around 2004, 2006, and 2008, respectively. MCS5 surveyed the cohort children at age 11 in their last year of primary school, mostly in 2012. The main informants were generally the biological mothers (99% at MCS1, 96% by MCS5). When possible, partners of the main respondent in two-parent families were also interviewed. For the most part, partners were the biological fathers (99% at MCS1).

From MCS1 to MCS5, 19,243 families have been interviewed at least once. Typical for a longitudinal study there has been loss to follow-up. Known predictors of non-response include younger and less educated parents, not breastfeeding, renting the home, moving home and various forms of partial response to previous surveys. Children included in this study have CP reported by the main respondent (hereafter, mother) on at least two ages. Our sample included one child per family, excluding those who were the second or third born in sets of twins and triplets. Hence, the final analyses were based 6458 boys and 6340 girls. Of these, 4769 boys and 4681 girls also had data from the partner of the main respondent (hereafter, father) at MCS1 for some of the measures, as indicated below.

### Measures

Table 1 shows descriptive statistics on the measures by gender.

**Table 1:** Gender differences among conduct problems and early risk factors

Measures	Boys		Girls			
	Mean(SD)	95% CI	Mean(SD)	95% CI	*F*-test	Cohen’s *d*
Probability of CP Age 3	0.22(0.41)	(0.21–0.24)	0.19(0.40)	(0.18–0.20)	*F*(1, 11,215) = 12.56***	0.07
Probability of CP Age 5	0.13(0.33)	(0.12–0.14)	0.08(0.29)	(0.07–0.09)	*F*(1, 11,712) = 43.06***	0.16
Probability of CP Age 7	0.13(0.33)	(0.12–0.14)	0.08(0.27)	(0.07–0.10)	*F*(1, 11,417) = 67.01***	0.17
Probability of CP Age 11	0.14(0.34)	(0.12–0.15)	0.09(0.28)	(0.08–0.10)	*F*(1, 12,797) = 50.66***	0.16
Risk factors						
Lowest household education	0.18(0.38)	(0.16–0.20)	0.18(0.38)	(0.16–0.20)	*F*(1, 12,333) = 0.02	
Lowest family income	0.23(0.42)	(0.21–0.25)	0.24(0.43)	(0.22–0.26)	*F*(1,12,355) = 0.04	
Single parenthood	0.18(0.38)	(0.16–0.19)	0.17(0.38)	(0.15–0.18)	*F*(1, 12,358) = 0.67	
Teenage mother	0.10(0.30)	(0.09–0.11)	0.10(0.30)	(0.09–0.11)	*F*(1, 12,358) = 0.29	
Smoked in pregnancy	0.27(0.44)	(0.25–0.29)	0.25(0.44)	(0.23–0.27)	*F*(1, 12,797) = 5.53	
Maternal depression	1.20(1.50)	(1.16–1.25)	1.19(1.54)	(1.15–1.24)	*F*(1, 11,990) = 0.12	
Maternal detachment	2.50(0.37)	(2.49–2.52)	2.45(0.37)	(2.44-2.46)	*F*(1, 12,359) = 4.19	
Maternal neglectful attitudes	1.26(0.39)	(1.25–1.28)	1.28(0.43)	(1.26–1.29)	*F*(1, 12,050) = 2.44	
Paternal neglectful attitudes	1.27(0.37)	(1.26–1.29)	1.27(0.38)	(1.25–1.28)	*F*(1, 9091) = 0.03	
Maternal partner relationship	2.04(0.66)	(2.02–2.06)	2.04(0.69)	(2.02–2.06)	*F*(1, 10,025) = 0.05	
Paternal partner relationship	2.07(0.58)	(2.05–2.10)	2.06(0.62)	(2.04–2.09)	*F*(1, 8899) = 0.38	
Infant delayed development	1.51(0.21)	(1.50–1.51)	1.46(0.21)	(1.45-1.47)	*F*(1, 12,351) = 93.53***	0.24
Infant difficult temperament	1.92(0.73)	(1.90–1.95)	2.04(0.80)	(2.02-2.07)	*F*(1, 12,048) = 52.54***	0.16

***p < 0.001

#### Conduct Problems

In MCS, CP at ages 3, 5, 7 and 11 were assessed by the Strengths and Difficulties Questionnaire (SDQ) (Goodman 1997, 2001). The SDQ is a screening questionnaire with extensive psychometric support (www.sdqinfo.com). In the MCS, construct, convergent, discriminant and predictive validity have been established for age 3, 5 and 7 SDQ subscales, showing good internal reliability with alphas ranging from 0.77 and 0.82 for CP (Croft et al. 2015). At age 11, the internal reliability of CP is acceptable (alpha = 0.68) (Flouri et al. 2017). In a self-completion instrument, mothers report on the child’s mental health symptoms in the past 6 months. The questionnaire assesses CP with five items: 1) “often has temper tantrums or hot tempers”; 2) “generally obedient, usually does what adults request” (reverse-coded); 3) “often fights with other children or bullies them”; 4) “often lies or cheats”; and 5) “steals from home, school or elsewhere.” Each item is marked as 0 = not true, 1 = somewhat true, or 2 = very true. In order to ensure that levels of CP are clinically meaningful, we used SDQ cut-off points based on the UK norms split by age bands (Meltzer et al. 2000), where the 10% of children with the highest scores were considered to be at high risk of CP (0 = not high risk; 1 = high risk).

#### Early Risk Factors

These were gathered at the MCS1 interview when the children were 9 months old. All measures were coded as risk factors. Categorical measures were dichotomized, with the category representing the highest level of risk coded as 1 and the other categories as 0. For continuous measures, higher scores represented higher levels of risk.

#### Low Parental Education

Both mothers and fathers (where present) reported their educational qualifications. If either had no UK qualifications (General Certificate Secondary of Education, A-levels, higher education or postgraduate degree) the risk factor takes the value of 1, which applied in 17% of cases (1 = no qualifications; 0 = some qualifications).

#### Low Family Income

Responses on the family’s total net income were categorised into equivalized income quintiles. The lowest quintile was treated as the risk factor (1 = lowest quintile; 0 = second through highest quintile).

#### Single Parenthood

Fifteen percent of mothers were neither married nor cohabiting with a partner at the first survey (1 = single mother; 0 = partner).

#### Teenage Mother

Ten percent of mothers were teenagers when the cohort child was born (1 = 19 years or younger; 0 = ≥20 years).

#### Smoked in Pregnancy

Mothers who reported smoking currently or within last two years were asked how much they had smoked just before they became pregnant and whether they changed the amount they smoked during their pregnancy with the cohort baby, and, if they had changed, the amount smoked per day after the change was made. The percentage of mothers (26%) smoking at some stage during their pregnancy is derived from these 3 variables (1 = smoked in pregnancy; 0 = did not smoke in pregnancy).

#### Maternal Depression (alpha = 0.72)

This is a 9-item count variable as reported in Johnson et al. (2015) derived from the (24 item) Malaise Inventory (Rutter et al. 1970). Mothers answered such questions as “everything gets on my nerves” and “I often feel miserable or depressed” (1 = yes; 0 = no).

#### Mother-Infant Detachment (alpha = 0.82)

A mean score of six items from the Condon Maternal Attachment Questionnaire was used to assess mother-to-infant attachment (Condon and Corkindale 1998). Mothers answered questions including, “When I am not with the baby, I find myself thinking about him” (reversed) and “When I am taking care of the baby, I have feelings of annoyance and irritation”, where 1 = very rarely; 4 = almost all the time.

#### Parental Neglectful Attitudes (alpha = 0.74 for Mothers, 0.66 for Fathers)

This scale was adapted from the Avon Longitudinal Study of Parents and Children (Golding et al. 2001). A mean score was calculated for mothers and fathers using three questions about how babies should be brought up such as, “cuddling a baby is very important” and “stimulation for development is important” (1 = strongly agree; 5 = strongly disagree).

#### Relationship Problems with Partner (alpha = 0.82 for Mothers, 0.77 for Fathers)

In families with a full-time resident partner, both mothers and fathers were asked 7 items from the Golombok Rust Inventory of Marital State (Rust et al. 1990). A mean score was calculated from the 7 questions including, “My partner does not seem to listen to me” and “I suspect we may be on the brink of separation” (1 = strongly disagree; 5 = strongly agree).

#### Infant Delayed Development (alpha = 0.64)

A mean score was calculated from mother reports including 8 questions from the Denver Developmental Screening Test to assess social and communication skills, as well as motor coordination typical for a 9 month old child such as “can sit up without being supported”, where 1 = often, 2 = once in a while, 3 = not yet (Frankenburg and Dodds 1967), and 5 questions from the MacArthur Communicative Development Inventories (CDI) to identify early communicative gestures such as “waves bye-bye”, where 1 = often, 2 = sometimes, 3 = not yet (Fenson et al. 1993).

#### Infant Difficult Temperament (alpha = 0.67)

Mothers answered 14 questions from the Carey Infant Temperament Scale, covering aspects of adaptability, mood and regularity, where 1 = almost never; 5 = almost always (Carey and McDevitt 1995). A mean score was calculated from items including “wary of strangers after 15 minutes” and “fretful in a new place or situation”.

### Statistical Analyses

The analyses were conducted in three stages using STATA 14. Given our interest in distinct gender-specific trajectories among females, we opted for the separate group analysis, using as our focal variable an indicator of clinically meaningful CP. The first stage involved identifying gender-specific pathways of CP using group-based trajectory modelling in STATA TRAJ (Jones and Nagin 2013). Developed by Nagin (2005), group-based trajectory modelling is a specialized form of finite mixture modelling which assumes that a population is composed of distinct groups, defined by their developmental trajectories (see Nagin and Odgers 2010). Full Information Maximum likelihood (FIML) was used for the estimation of the model parameters, thereby including every case with at least two maternal ratings (Schafer and Graham 2002). Binary logit distribution was specified as CP are considered a dichotomous variable (e.g., whether clinically meaningful or not). Trajectories were modelled as a function of each child’s age measured in months at each interview. The best fitting solution was selected using the criteria defined by Nagin (2005) including consideration of the most parsimonious solution, interpretability, and indicators of model fit, such as the Bayesian Information Criterion (BIC), average posterior probability of group membership (0.70 being acceptable for all groups) and a close correspondence between the estimated probability of group membership and the proportion assigned to that group-based on the posterior probability of group membership.

In order to account for the complex survey design of MCS, we used the *svy* prefix in STATA for the following stages of the analyses. The *svy* prefix is designed for use with complex survey data and provides for sampling weights, one or more stages of clustered sampling, and stratification. Attrition weights were also applied, developed by the MCS survey team to correct for biases due to non-response (**Hansen** 2014, 18–22). Individual respondents are given an inverse probability weight of response at each wave according to characteristics at the previous wave. When multiplied across waves and by the original sampling weight, this aims to restore the social profile of the original population.

First, we tested for gender differences in CP and the early risk factors using univariate regressions for each predictor on gender. To account for the number of tests, we applied the Bonferroni correction. We thus only report those differences where *p* < 0.001. For significant differences, the effect size (using Cohen’s *d* which is the appropriate effect size measure if two groups have similar standard deviations and are of similar size) is reported. For the univariate analyses of the risk factors, we first fitted regressions for each predictor on trajectory group status, separately for boys and girls. We then conducted post-hoc tests to compare all possible pairwise differences among the four groups, using the Bonferroni correction to adjust for multiple comparisons. The range of effect sizes (using Hedges’ *g* which provides a measure of effect size weighted according to the relative size of each sample) is reported for significant pairwise differences. For the multivariate analyses, we ran multinomial logistic regressions separately for the boys and girls. For Parental Neglectful Attitudes, families where both mothers and fathers data were available, the mean of their mean scores was used in the multivariate analyses. Relationship Problems with Partner was only assessed in families where a full-time resident partner was present, thereby excluding single parents, thus was not included in the multivariate analyses.

## Results

### Gender Differences in CP and Early Risk Factors

As expected, boys in our sample had, on average, higher rates of clinically meaningful CP than did girls at all four points in early to mid-childhood (Table 1). The highest rates were at age 3 (22% of boys, 19% of girls), when the gap between them was narrowest. Between ages 5 and 7, the average prevalence rates levelled off, at 13% for boys and 8% for girls, each rising by one percentage point at age 11, to 14% and 9% respectively. Table 1 also shows the risk factors at 9 months that we included in our models, for boys and girls. Mostly there were no significant differences between them, at *p* < 0.001, apart from boys showing more delayed development but less difficult temperament than girls. For those measures showing statistically significant gender differences, their small effect sizes suggest substantial within-gender heterogeneity.

### Trajectories of CP

We examined models with three to five trajectories with linear to cubic functional forms, separately by gender (*N* = 6458 boys and 6340 girls). For both, the four group model fit the data best (Nagin 2005), with the lowest BIC score compared to the three and five group models, the average posterior probability being higher than 0.70 and a close correspondence between the estimated probability of group membership and the proportion assigned to that group based on the posterior probability of group membership. Of the four groups, there was a low group, along with three groups showing childhood-onset CP. In order to distinguish among the age of onset, we labelled the childhood-onset groups as ‘early-onset, persistent’, ‘early-onset, desisting’ and ‘school-onset’. The three-group solution excluded the ‘school-onset’ group, while the five-group model included an additional persistent, moderate group, suggesting that the four group solution provides the most parsimonious summary of distinct patterns. Although the trajectories were given the same labels, the key outputs (i.e., shape of trajectory, proportion of population belonging to each trajectory, posterior probability and the shape of the model with the maximized BIC) were different for girls and boys, as discussed below.

**Fig. 1:**
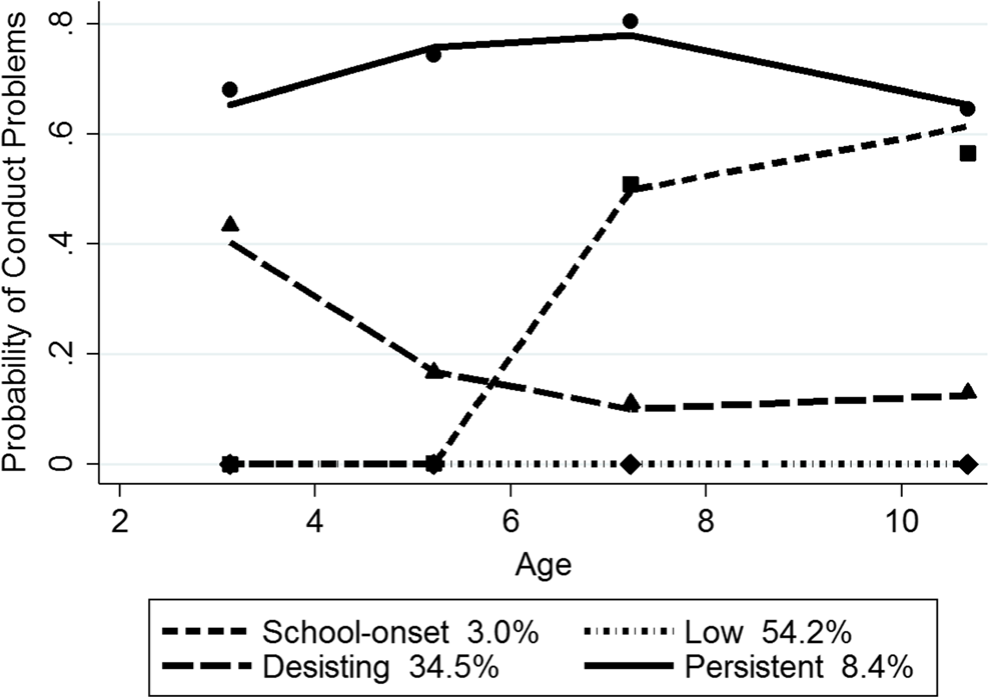
Boys’ trajectory groups. Note. Shown are estimated trajectories (lines), observed group means at each age (markers) and estimated group percentages

For boys, the BIC score for the four group model (−8107.69) was better than for the three (−8121.99) and five (−8123.25) group models. The mean posterior probability scores, indexing the degree to which each boy fits his assigned trajectory, ranged from 0.76 to 0.86 for the four trajectory model, with a mean of 0.79, indicating that most boys fit their assigned trajectory well. Figure 1 depicts the probability of clinically relevant CP for the four trajectory groups in boys from ages 3 to 11, along with the estimated proportion in each group (total N of observation occasions = 23,706). As shown in Fig. 1, the predicted and observed means are close, indicating a good fit of the model. Furthermore, estimated and actual proportions of each group were similar. The ‘low problem’ group (54.2% estimated; 56.2% actual) showed a stable trajectory close to zero. The ‘early-onset, desisting’ group (34.5% estimated, 33.3% actual) had a moderate probability of CP in early childhood (around 40%), declining from ages 3 to 5 and a low, stable probability of CP thereafter around 10%. The ‘early-onset, persistent’ group (8.4% estimated; 7.6% actual) followed a high probability of CP, which peaked slightly at age 7 but was still over 60% at age 11. In the ‘school-onset’ group, a small percentage of boys (3.0% estimated; 2.9% actual) displayed a low probability of CP at ages 3 and 5, with a substantial increase by age 7, rising to nearly 60% at age 11.

**Fig. 2:**
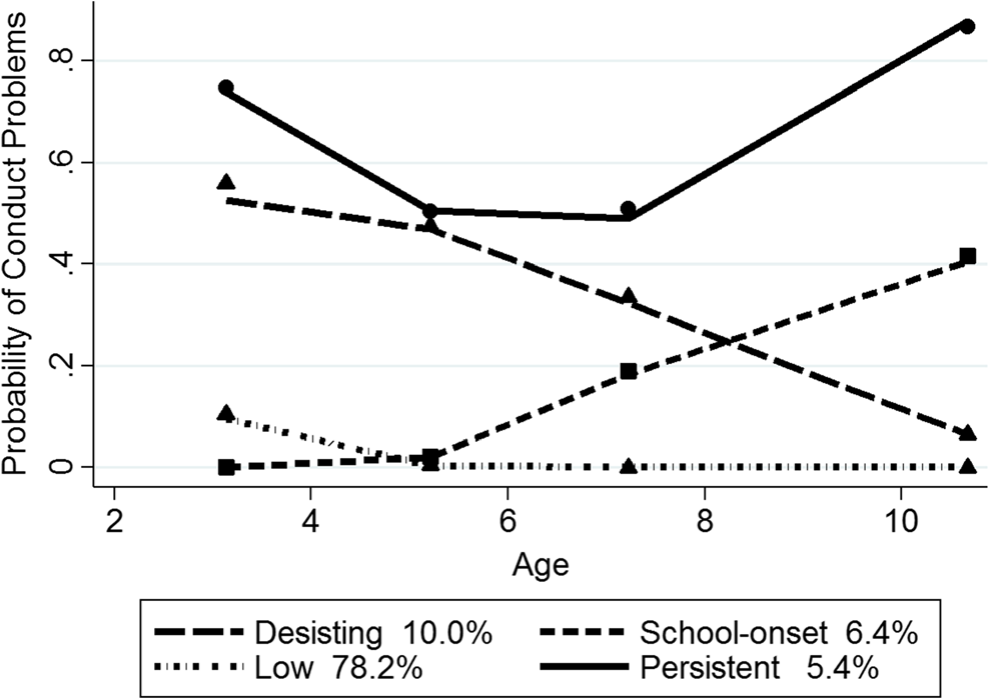
Girls’ trajectory groups. Note. Shown are estimated trajectories (lines), observed group means at each age (markers) and estimated group percentages

For girls, the BIC score for the four group model (−6386.31) was better than for the three (−6414.97) and five (−6406.30) group models. The mean posterior probability scores for girls ranged from 0.71 to 0.92 for the four-trajectory model, with a mean of 0.82, indicating that most girls fit their assigned trajectory well. Figure 2 depicts the probability of clinically relevant CP for the four trajectory groups in girls from ages 3 to 11, along with the estimated percentage in each group (total N of observation occasions = 23,436). As shown in Fig. 2, the predicted and observed values have a high level of correspondence, indicating a good fit of the model. Furthermore, estimated and actual proportions of each group were similar. The ‘low’ group (78.2% estimated; 79.6% actual) showed a small probability of CP at age 3, around 10%, that decreased to close to zero from age 5 onwards. As expected, and as discussed below, there were more girls on a low pathway than there were boys. The ‘early-onset, desisting’ group (10% estimated; 9.2% actual) displayed a high probability of CP at age 3 (close to 60%) that declined sharply to around 5% at 11, but they are a considerably smaller group than the analogous group of boys. The ‘early-onset, persistent’ group (5.4% estimated; 4.9 actual) displayed a probability of CP almost 80% at age 3, declining slightly to age 5 and then sharply increasing from age 7. This small group of girls showed a probability of CP around 80% at both ages 3 and 11. The ‘school-onset’ group (6.4% estimated; 6.3% actual) showed a low probability of CP from ages 3 to 5 that gradually increased from ages 5 to 11 but only to 40%.

### Univariate Early Risk Factors for Boys and Girls

Tables 2 and 3 present the univariate early risk factors of each trajectory for boys and girls, respectively. For boys, there were significant differences (*p* < 0.001) among the CP trajectories on all risk factors considered, with the exception of Infant Difficult Temperament and, for girls, everything other than the Teenage Mother, Parental Neglectful Attitudes and Infant Delayed Development. Superscripts reflect post-hoc analyses, where all possible pairwise differences among the four groups were compared. Significant differences are shown for those groups who do not share any particular superscripts. For both genders, the pre- and post-natal factors at 9 months that one might expect to be favourable did indeed differentiate the ‘low’ group from those going on to display the various patterns of CP, with small effects for parenting variables and medium effects for socio-economic factors and maternal psychopathology. The greatest contrast is shown between those on the ‘low’ pathway and those on ‘early-onset, persisting’ pathway, with ‘school-onset’ and ‘early-onset, desisting’ paths showing similar intermediate values, for the most part, especially for boys.

**Table 2:** Mean differences in early risk factors according to trajectory group for boys

Risk factors	Low (54.2%)	School-age onset (3%)	Early-onset, desisting (34.5%)	Early-onset, persistent (8.4%)		
	Mean(SD)	Mean(SD)	Mean(SD)	Mean(SD)	*F*-Test	Hedges’ *g*
Lowest household education	0.14(0.35)^a^	0.28(0.41)^b, c^	0.23(0.42)^b^	0.33(0.43)^c^	*F* (3, 6229) = 22.19***	0.24–0.53
Lowest family income	0.17(0.38)^a^	0.32(0.42)^b^	0.32(0.46)^b^	0.44(0.45)^b^	*F*(3, 6229) = 41.76***	0.36–0.69
Single parenthood	0.13(0.34)^a, b^	0.19(0.36)^b^	0.24(0.42)^b, c^	0.34(0.44)^c^	*F* (3, 6229) = 26.98***	0.36–0.59
Teenage mother	0.07(0.27)^a^	0.06(0.22)^a^	0.13(0.33)^b^	0.19(0.35)^b^	*F(*3, 6455) = 11.59***	0.19–0.43
Smoked in pregnancy	0.24(0.44)^a^	0.41(0.45)^b^	0.40(0.48)^b^	0.53(0.46)^c^	*F* (3, 6455) = 53.92***	0.35–0.66
Maternal depression	0.98(1.37)^a, b^	1.32(1.31)^b, c^	1.55(1.64)^c^	2.01(1.70)^d^	*F* (3, 6047) = 66.06***	0.48–0.73
Maternal detachment	3.47(0.37)^a^	3.62(0.38)^b^	3.55(0.37)^b^	3.59(0.39)^b^	*F* (3, 6072) = 16.86***	0.22–0.40
Maternal neglectful attitudes	1.24(0.38)^a^	1.31(0.45)^b, c^	1.30(0.41)^b, c^	1.37(0.42)^c^	*F* (3, 6081) = 15.42***	0.15–0.34
Paternal neglectful attitudes	1.25(0.37)^a^	1.34(0.38)^b^	1.31(0.38)^b^	1.33(0.36)^b^	*F*(3, 4586) = 7.39***	0.16–0.22
Maternal partner relationship	1.96(0.64)^a^	2.25(0.59)^b, c^	2.19(0.69)^b^	2.35(0.64)^c^	*F*(3, 5041) = 33.38***	0.35–0.61
Paternal partner relationship	2.03(0.59)^a^	2.19(0.51)^b^	2.17(0.60)^b^	2.19(0.56)^b^	*F*(3, 4588) = 12.28***	0.24–0.27
Infant delayed development	1.50(0.21)	1.54(0.19)	1.50(0.20)	1.54(0.22)	*F* (3, 6225) = 3.75	
Infant delayed temperament	1.90(0.73)	2.01(0.65)	1.98(0.75)	1.93(0.68)	*F* (3, 6078) = 2.77	

Post-hoc analyses using Bonferroni’s method identified significant pairwise comparisons (*p* < 0.05) between groups, shown when group means do not share any similar superscripts

***p < 0.001

**Table 3:** Mean differences in early risk factors according to trajectory group for girls

Risk factors	Low (78.2%)	School-age onset (6.4%)	Early-onset, desisting (10%)	Early-onset, persistent (5.4%)		
	Mean(SD)	Mean(SD)	Mean(SD)	Mean(SD)	*F*-Test	Hedges’ *g*
Lowest household education	0.16(0.37)^a^	0.24(0.42)^b^	0.29(0.44)^b^	0.37(0.45)^c^	*F* (3, 6123) = 22.57***	0.21–0.56
Lowest family income	0.22(0.42)^a^	0.36(0.47)^b^	0.35(0.46)^b^	0.44(0.47)^b^	*F*(3, 6127) = 19.78***	0.31–0.52
Single parenthood	0.15(0.36)^a^	0.26(0.43)^b^	0.25(0.42)^b^	0.27(0.42)^b^	*F* (3, 6339) = 45.02***	0.33
Teenage mother	0.08(0.27)	0.13(0.33)	0.15(0.34)	0.17(0.35)	*F*(3, 6339) = 2.72	
Smoked in pregnancy	0.25(0.44)^a^	0.37(0.47)^b^	0.44(0.48)^b^	0.50(0.47)^b^	*F* (3, 6340) = 32.29***	0.27–0.57
Maternal depression	1.06(1.43)^a^	1.65(1.72)^b^	1.87(1.78)^b^	2.03(1.70)^b^	*F* (3, 5943) = 40.64***	0.41–0.67
Maternal detachment	2.44(0.36)^a, b^	2.48(0.38)^b^	2.52(0.42)^b, c^	2.52(0.37)^c^	*F* (3, 6124) = 6.04***	0.22
Maternal neglectful attitudes	1.26(0.42)	1.30(0.40)	1.34(0.44)	1.34(0.42)	*F* (3, 5969) = 3.44	
Paternal neglectful attitudes	1.26(0.37)	1.30(0.40)	1.29(0.37)	1.34(0.38)	*F* (3, 4502) = 1.97	
Maternal partner relationship	2.00(0.67)^a^	2.18(0.68)^b^	2.25(0.73)^b,c^	2.46(0.78)^c^	*F*(3, 4984) = 16.89***	0.37–0.68
Paternal partner relationship	2.05(0.61)^a^	2.16(0.64)^b^	2.19(0.62)^b^	2.17(0.58)^b^	*F*(3, 4407) = 4.79***	0.20
Infant delayed development	1.46(0.21)	1.48(0.23)	1.48(0.22)	1.47(0.23)	*F* (3, 6126) = 0.72	
Infant difficult temperament	2.02(0.77)^a, b^	2.12(0.86)^b, c^	2.19(0.85)^c^	2.18(0.80)^c^	*F* (3, 5971) = 6.23***	0.22

Post-hoc analyses using Bonferroni’s method identified significant pairwise comparisons (*p* < 0.05) between groups, shown when group means do not share any similar superscripts

***p < 0.001

### Multivariate Models of Early Risk Factors for Boys and Girls

The multinomial estimates contrast the chances, for each gender, of being in one of the three childhood-onset CP trajectories relative to the ‘low’ group. Table 4 shows 6 sets of relative risk ratios and their 95% confidence intervals. Since the variables have been defined in a direction likely to raise risks, we are generally looking for estimates of RRR greater than 1, with lower bounds on their confidence intervals also over this threshold. Table 4 demonstrates that where at least one parent had no qualifications children showed highly significant risks of being on either of the two ‘early-onset’ trajectories relative to where parents (each) had at least some qualification. Family income in the bottom quintile significantly raised the chances of being on the ‘early-onset, persistent’ trajectories for boys and girls. Single parenthood at 9 months was significantly associated with the early-onset paths for boys, but not with any of the girls’ trajectories. Although there were significant differences in univariate analysis, teenage motherhood showed no independent association with any of the CP groups for either boys or girls, given the inclusion of related socio-demographic variables. ‘School-onset’ generally was not significantly associated with the socio-demographic characteristics at first survey for either boys or girls.

**Table 4:** Multinomial regression model for boys and girls with relative risk ratios and 95% confidence intervals

Risk factors reported at 9 months	School-age onset Boys		Early-onset, desisting Boys		Early-onset, persistent Boys		School-age onset Girls		Early-onset, desisting Girls		Early-onset, persistent Girls	
	RRR	CI	RRR	CI	RRR	CI	RRR	CI	RRR	CI	RRR	CI
Low household education	1.69	(0.91,3.13)	1.33**	(1.07,1.64)	1.92***	(1.47,2.52)	1.38	(0.94,2.02)	1.64**	(1.19,2.24)	2.01***	(1.41,2.88)
Low family income	1.52	(0.71,3.26)	1.23	(0.97,1.57)	1.46*	(1.00,2.13)	1.49	(0.96,2.32)	1.09	(0.74,1.62)	1.66**	(1.09,2.54)
Single parenthood	1.12	(0.48,2.59)	1.45**	(1.14,1.85)	1.84***	(1.25,2.70)	1.36	(0.85,2.18)	1.23	(0.83,1.83)	1.04	(0.63,1.73)
Teenage mother	0.50	(0.17,1.48)	1.12	(0.82,1.55)	1.33	(0.89,1.98)	0.92	(0.38,2.25)	1.42	(0.80,2.52)	1.21	(0.65,2.23)
Smoked in pregnancy	2.34**	(1.24,4.42)	1.81***	(1.51,2.17)	2.58***	(1.97,3.37)	1.30	(0.87,1.94)	1.98***	(1.50,2.61)	2.18***	(1.54,3.09)
Maternal depression	1.02	(0.87,1.19)	1.19***	(1.13,1.25)	1.32***	(1.23,1.43)	1.20***	(1.09,1.33)	1.25***	(1.17,1.33)	1.29***	(1.19,1.41)
Maternal detachment	2.46**	(1.35,4.49)	1.36**	(1.11,1.67)	1.49*	(1.09,2.06)	1.07	(0.70,1.61)	1.31	(0.94,1.85)	1.25	(0.84,1.86)
Parental neglectful attitudes	1.56	(0.84,2.89)	1.37**	(1.10,1.72)	1.86***	(1.30,2.67)	1.05	0.69,1.61)	1.06	(0.76,1.48)	1.08	(0.72,1.61)
Infant delayed development	2.09	(0.62, 7.04)	1.14	(0.80,1.62)	2.79***	(1.57,4.95)	1.64	(0.73,3.66)	1.34	(0.77,2.36)	1.06	(0.44,2.59)
Infant difficult temperament	1.23	(0.73, 2.10)	1.27**	(1.08,1.49)	1.09	(0.84,1.42)	1.19	(0.83,1.70)	1.63***	(1.26,2.09)	1.50*	(1.07,2.07)

***p ≤ 0.001, **p ≤ 0.01, *p ≤ 0.05; *N* = 6023 (boys); 5934 (girls)

In terms of the maternal characteristics, smoking in pregnancy emerged as a significant predictor of all three childhood-onset CP pathways for boys and both early-onset tracks for girls. Maternal depression raised the estimates with the exception of the ‘school-onset’ trajectory for boys. Low maternal attachment raised the chances of boys being on childhood-onset CP trajectories (particularly the ‘school-onset’ trajectory), but was not related to the girls’ pathways. Parents’ neglectful attitudes (which incorporates the father’s views where reported) was significantly associated with boys being on the early-onset paths, particularly the ‘early-onset, persistent’ path, but was unrelated to the girls’ CP trajectories.

For infant characteristics, developmental delay, with a higher incidence in boys, was significantly associated with an increased chance of their being on the ‘early-onset, persistent’ pathway, but showed no associations for girls. A difficult temperament at 9 months was associated with the ‘early-onset, desisting’ and ‘early-onset, persistent’ pathways among girls. For boys, those with more difficult temperaments were more likely to be on the ‘early-onset, desisting’ pathway than the ‘low’ problems track.

## Discussion

Using a developmental-pathways approach, our overall aim was to identify gender typical trajectories of childhood-onset CP and to see if risk factors already present in infancy discriminate among the different pathways. Based on data collected for a nationally representative cohort study, we provide evidence of diversity in childhood-onset pathways of CP among girls as well as boys. We used group-based trajectory modelling to capture the heterogeneity in the development of childhood-onset CP over a 9-year period, between ages 3 and 11. We identified four distinct CP trajectories among boys and girls including a low group and three groups showing childhood-onset CP. Although the trajectories were given the same labels (‘low’, ‘early-onset, persistent’, ‘early-onset, desisting’ and ‘school-onset’), they take somewhat different shapes and have different prevalence rates for boys and girls.

Our findings indicate that girls generally have fewer CP and the ‘low’ pathway is more prevalent among girls than boys (78% versus 54%). The rate of children in the ‘low’ trajectory is similar to other UK studies, who report around 65% of children as consistently low from early childhood (Barker and Maughan 2009). We also find more boys than girls who are on an ‘early-onset, persisting’ trajectory (8% versus 5%). Further, our rates in the ‘early-onset, persistent’ trajectories are similar to prior evidence from international community samples (e.g., Broidy et al. 2003; Côté et al. 2006; Van Lier et al. 2005). We also note that boys in this trajectory appear to be more or less persistently high from age 3. Girls show similarly high levels at age 3, then there is a dip between ages 5 and 7, followed by a spurt at age 11, which may be associated with the onset of puberty.

We also identified an initially high at age 3 and then desisting trajectory for both males and females, comprising 34% of males and 10% of females. This finding contrasts with other studies of this age group (Campbell et al. 2010; Côté et al. 2001), suggesting that a non-negligible number of females show both early-onset, persistent *and* early-onset, desisting pathways of CP. As these studies are from school-age, they may have missed identification of an ‘early-onset, desisting’ group, who might have otherwise been labelled as ‘low’ by age 6. However, the prevalence of desisting girls in our study coincides with other research also reporting a desisting trend for females (Barker and Maughan 2009; Fontaine et al. 2008). In line with these studies, our females begin to desist around age 7. In contrast to research on aggressive behaviour (Hay et al. 2000; Keenan and Shaw 1997), however, the more numerous boys in the ‘early-onset, desisting’ track appear to desist at an earlier age (soon after age 3) than girls.

In addition, we identified a distinct trajectory of ‘school-onset’ comprising 3% of boys and 6% of girls. Among boys, the ‘school-onset’ pathway shows a sharper increase from age 7 and seems to lead to higher prevalence of CP at 11 than among girls. The ‘school-onset’ trajectory has not been identified in previous studies of this age group using a developmental-pathways approach, perhaps due to variations in the measurement of CP, the timing of assessments or the use of different modelling techniques. Given that many previous studies began data collection at school-age or older, a ‘school-onset’ group may be labelled as ‘early-onset, persisting’ in these previous studies. Our data on age three thus suggest a period of high risk for raised CP for both genders, with greater heterogeneity in childhood-onset CP pathways than previously found using samples assessed from school-age.

Focusing on the results for the early predictor variables in the multivariate models, our findings suggest that ‘school-onset’ is not significantly associated with initial socio-economic disadvantage, in contrast to the early-onset trajectories, but with maternal factors, such as smoking in pregnancy and maternal detachment among boys and maternal depression among girls. ‘School-onset’ appears to be equally prevalent among boys and girls from advantaged and disadvantaged family backgrounds and maternal characteristics seem to be a significant risk factor for both girls and boys. However, there appear to be gender differences regarding which aspects matter, suggesting gender-specific sensitivities.

Regarding early-onset CP, we find that low parental education, smoking in pregnancy and maternal depression are significant risk factors for both boys and girls - and for both desisting and persisting patterns. Reduction of these risk factors might go a long way in preventing the early-onset of CP. As shown by Barker and Maughan (2009), we also find that the early-onset, persisting and desisting trajectories could be differentiated by the range and severity of these risks. For example, among boys the RRR associated with low parental education is 1.92 for ‘early-onset, persistent’ CP and 1.33 for ‘early-onset, desisting’ CP in comparison to those on the ‘low’ pathway (among girls it is 2.01 versus 1.64).

A distinct risk factor for the ‘early-onset, persistent’ CP trajectory for both girls and boys is low family income suggesting that this pathway is more strongly associated with socio-economic disadvantage than the early-onset, desisting trajectory. For boys, delayed infant development, among whom it is more prevalent, also distinctly predicts the ‘early-onset, persisting’ CP pathway. Early risk factors for early-onset, persisting and desisting CP trajectories among boys compared to girls are having a single parent, maternal detachment and a neglectful attitude among parents. Among both boys and girls, a difficult temperament is associated with the ‘early-onset, desisting’ trajectory and further predicts the ‘early-onset, persistent’ CP trajectory among girls. These findings suggest that boys might be (on average) more sensitive and require more maternal attention during early childhood than girls (Kraemer 2000). It has also been argued that in boys the formation of secure caregiver attachment is more strongly influenced by parental unavailability or insensitivity than in girls (Cicchetti and Tucker 1994). It might also be possible that among boys a difficult early temperament could be a protective factor, inviting more parental attention and preventing persistence of early signs of CP.

### Limitations

There are a number of limitations to bear in mind. First, CP and many of the risk factors were assessed on maternal reports, raising the problem of informant and methodological biases. Future studies should include multiple informants and biological indicators, if possible. Second, the extent of our analyses were limited by the measures included in the multi-purpose longitudinal survey of a national cohort, many of which relied on a parsimonious measurement strategy. As a result, some of our measures, such as those measuring infant development, had low alphas below 0.70, which may compromise their reliability. Furthermore, the SDQ relies on a narrowly defined pool of overt and covert items of CP, which may limit our ability to detect gender variations among pathways. The SDQ does not assess relational aggression, for example, which may explain the predominance of adolescent-onset in antisocial girls (Frick and Viding 2009). Future studies, especially those that extend into the adolescent period, should consider whether the inclusion of relational aggression and other facets of CP provide more sensitivity in detecting potential gender differences among developmental pathways of CP. Third, as in all longitudinal studies, we were faced with the problem of missingness in the data due to non-response to certain items or for a whole wave of data collection. We addressed the problem using MCS attrition weights and FIML estimation as implemented in STATA to adjust the likelihood function so that each case contributes information on the variables that are observed. Fourth, group-based trajectory analysis only provides a descriptive summary of a potential underlying typology in pathways. The fit indicators provide some guidelines about the number of types to select, and the final selection is based on consideration of parsimony, the interpretability, the BIC statistics and average posterior probability of group membership. Four classes were found in other longitudinal studies, while others established three or five classes. Fifth, running the analysis separately by gender avoids the potential misclassification of girls with their generally lower levels of CP. On the other hand, this analytic strategy does not allow one to test gender differences statistically in the trajectories of CP or their covariates. Sixth, we have only assessed a subset of precursors of CP pathways, focusing on the pre- and post-natal period up to age 9 months. This approach enables the identification of earliest risk markers in pregnancy and infancy for the purposes of prevention, but omits other factors measured in childhood such as callous-unemotional traits and impulsivity, which may go some way in further distinguishing the childhood-onset pathways between boys and girls (see Frick and Viding 2009). Furthermore, since the measurement of maternal depression, parenting attitudes and child development is at least two years before the evidence on CP, we cannot account for possible reciprocal difficulties between parents and offspring. We also do not assess the effect of changes in risk factors on CP development. For example, if the family composition, income or mental health situations deteriorated, this might help account for the ‘school-onset’ cases, given the strong association of such factors with early-onset CP. Lastly, we only followed our sample up to age 11 and thus do not have evidence whether childhood-onset problems persisted beyond early adolescence. Our study is thus also not well suited for identification of the adolescent-onset trajectory.

### Conclusions and Implications

Our findings go some way toward further distinction of childhood-onset CP pathways and their earliest predictors among boys and girls. Our results suggest that a non-negligible number of girls (5%) follow the ‘early-onset, persistent’ CP trajectory starting at age 3 and are potentially at an increased risk for continued adjustment problems in adolescence and adulthood. Moreover about 10% of girls in our sample show an ‘early-onset, desisting’ CP trajectory, and about 6% manifest a ‘school-onset’ CP trajectory. The taxonomic framework developed to conceptualise the common antecedents, correlates and outcomes of childhood-onset CP among males (Moffitt 2006) thus also seems to be applicable to females (see also Brennan and Shaw 2013), though needing some revision to capture apparent heterogeneity identified during early and middle childhood.

For both boys and girls, our findings suggest a strong association of social disadvantage in the first year of life with early-onset CP, particularly for problems that persist. Maternal depression and smoking in pregnancy also appear to be significant risk factors for both girls and boys. However, the results highlight some gender-specific precursors; with boys’ early-onset CP having a stronger association with their own delayed development and parenting factors than girls’. Our findings suggesting boys’ greater sensitivity to parenting risk factors correspond with recent research findings showing that boys from disadvantaged homes are both more sensitive to parenting skills and responsive to intervention than are girls (Heckman et al. 2017).

In terms of clinical and service implications, our findings highlight the importance of targeting high-risk mothers from pregnancy. Given that socioeconomic deprivation was the single precursor distinguishing the early-onset, persistent CP pathway, poverty may represent an important factor for targeting high-risk mothers. Clinical interventions directed at reducing exposure to smoking and poor mental health of mothers appear to be especially promising, as would be measures aiming to improve maternal education and parent training. Prevention trials targeting childhood-onset CP may thus focus on these early indicators of risk– and maybe also aim to reduce uninvolved parenting attitudes and behaviours which were distinctly associated with the childhood-onset CP pathways among boys.

Future research should continue to examine the developmental mechanisms that underlie the continuities and discontinuities in these diverse trajectories, particularly those that distinguish early-onset, desisting boys and girls from those who persist on high CP trajectories. Also important is further investigation of ‘school-onset’ CP to gain a greater understanding of possible factors in the school context which may predict and protect children on that particular pathway. Such information has important implications for the timing, targeting and implementation of effective prevention programs tailored specifically for boys and girls within the different developmental CP pathways.
